# Quantitative MRI evaluation of glaucomatous changes in the visual pathway

**DOI:** 10.1371/journal.pone.0197027

**Published:** 2018-07-09

**Authors:** Mana Fukuda, Kazuko Omodaka, Yasuko Tatewaki, Noriko Himori, Izumi Matsudaira, Koji M. Nishiguchi, Takaki Murata, Yasuyuki Taki, Toru Nakazawa

**Affiliations:** 1 Department of Ophthalmology, Tohoku University Graduate School of Medicine, Sendai, Japan; 2 Department of Ophthalmic Imaging and Information Analytics, Tohoku University Graduate School of Medicine, Sendai, Japan; 3 Institute of Development, Aging and Cancer, Tohoku University Graduate School of Medicine, Sendai, Japan; 4 Department of Advanced Ophthalmic Medicine, Tohoku University Graduate School of Medicine, Sendai, Japan; 5 Diagnostic Radiology, Tohoku University Graduate School of Medicine, Sendai, Japan; 6 Department of Retinal Disease Control, Ophthalmology, Tohoku University Graduate School of Medicine, Sendai, Japan; Massachusetts Eye & Ear Infirmary, Harvard Medical School, UNITED STATES

## Abstract

**Background:**

The aims of this study were to investigate glaucomatous morphological changes quantitatively in the visual cortex of the brain with voxel-based morphometry (VBM), a normalizing MRI technique, and to clarify the relationship between glaucomatous damage and regional changes in the visual cortex of patients with open-angle glaucoma (OAG).

**Methods:**

Thirty-one patients with OAG (age: 55.9 ± 10.7, male: female = 9: 22) and 20 age-matched controls (age: 54.9 ± 9.8, male: female = 10: 10) were included in this study. The cross-sectional area (CSA) of the optic nerve was manually measured with T2-weighed MRI. Images of the visual cortex were acquired with T1-weighed 3D magnetization-prepared rapid acquisition with gradient echo (MPRAGE) sequencing, and the normalized regional visual cortex volume, i.e., gray matter density (GMD), in Brodmann areas (BA) 17, 18, and 19, was calculated with a normalizing technique based on statistic parametric mapping 8 (SPM8) analysis. We compared the regional GMD of the visual cortex in the control subjects and OAG patients. Spearman’s rank correlation analysis was used to determine the relationship between optic nerve CSA and GMD in BA 17, 18, and 19.

**Results:**

We found that the normal and OAG patients differed significantly in optic nerve CSA (p < 0.001) and visual cortex GMD in BA 17 (p = 0.030), BA 18 (p = 0.003), and BA 19 (p = 0.005). In addition, we found a significant correlation between optic nerve CSA and visual cortex GMD in BA 19 (r = 0.33, p = 0.023), but not in BA 17 (r = 0.17, p = 0.237) or BA 18 (r = 0.24, p = 0.099).

**Conclusion:**

Quantitative MRI parametric evaluation of GMD can detect glaucoma-associated anatomical atrophy of the visual cortex in BA 17, 18, and 19. Furthermore, GMD in BA 19 was significantly correlated to the damage level of the optic nerve, as well as the retina, in patients with OAG. This is the first demonstration of an association between the cortex of the brain responsible for higher-order visual function and glaucoma severity. Evaluation of the visual cortex with MRI is thus a very promising potential method for objective examination in OAG.

## Introduction

Glaucoma is the second most common cause of blindness worldwide, and the most common cause of vision loss in Japan. In glaucoma, axonal degeneration of the retinal ganglion cells (RGCs), mainly occurring in the lamina cribrosa, and thinning of the optic disc rim, in areas corresponding to retinal nerve fiber layer defects (RNFLDs), ultimately lead to RGC death. [1] Conventional glaucoma diagnosis relies on the confirmation of simultaneous functional loss and structural changes, evaluated with visual field testing and optical coherence tomography (OCT), respectively. The only evidence-based treatment for glaucoma is the reduction of intraocular pressure (IOP). [2] However, glaucoma is generally a chronic, progressive disease and even with successfully lowered IOP, glaucoma progress cannot be completely stopped in all cases. [3] Furthermore, in Asia, the relative prevalence of normal-tension glaucoma (NTG) [4] makes IOP measurement insufficient for glaucoma screening. Therefore, visual field testing is the primary method in Asia, not only for diagnosing glaucoma, but also for detecting progression. However, this is complicated by the fact that elderly patients are more susceptible to glaucoma. For example, in the Tajimi Study, the prevalence of POAG increased with age in a Japanese study population, [4] and Ernest PJ reported that age was one of the factors most clearly associated with glaucomatous visual field progression. [5] It can be difficult in elderly patients to obtain reliable data with visual field testing, which is subjective, or with existing objective examinations, such as fundus photography and OCT. Thus, new, objective methods of evaluating glaucoma severity are necessary.

In recent years, it has been conclusively shown that glaucoma causes damage not only to the eye itself, but also to the entire central visual pathway in the brain. Gupta et al. examined post-mortem tissue samples taken from patients with glaucoma, and found that the optic nerve, lateral geniculate nucleus, and visual cortex were obviously atrophied compared to patients without glaucoma. [6–8] The authors concluded that in cases of advanced glaucoma (with at least 50% visual field loss), there is distinct neurological damage in multiple area of the brain related to vision. Recent advancements in neuroimaging technologies have also shown that the central visual pathway degenerates in glaucoma patients, indicating that glaucoma is pathologically similar to other central nervous system neurodegenerative disorders. [9,10] Vision-related brain atrophy in patients with POAG has been found on both sides of the visual cortex and in the frontoparietal cortex, hippocampus, and cerebellar cortex. [11] Thus, the entire visual pathway, from the RGCs to the visual cortex, is involved in the pathophysiology of glaucoma.

Magnetic resonance imaging (MRI) is a useful method of detecting central nervous system changes, such as the ones mentioned above. MRI analysis has made remarkable progress in recent years. New imaging modes are especially relevant, because they can detect the movement of water molecules in the nerve fibers and axons. Diffuser tensor imaging (DTI) allows the imaging of the nerve fibers and the determination of nerve fiber quantity, which can reveal early axonal damage more sensitively than ordinary MRI. Functional MRI techniques, such as blood oxygen level dependent (BOLD) contrast imaging, are commonly used to detect ischemic changes and changes in the activity of the brain. Additionally, recent advanced imaging methods can evaluate the thickness of the brain cortex and the volume of the brain precisely and quantitatively with high-resolution 3D anatomical imaging. Furthermore, voxel-based spatial-normalization methods for MRI are enabled by the use of appropriate software, such as Statistic Parametric Mapping (SPM). Normalization compensates for individual differences in brain geometry, allowing the analysis of absolute regional cortical volume (i.e., gray matter density; GMD) in each voxel. GMD is a biomarker of brain function and allows the quantitative assessment of the location and degree of structural changes in the brain, such as those that are associated with normal aging and with neuro-degenerative diseases (e.g., Alzheimer’s disease). This type of GMD-based analysis, which is valid for both inter-subject and intra-subject comparisons, is termed voxel-based morphometry (VBM). Thus, recent advances in quantitative evaluation methods of brain structure based on MRI have made MRI more common in brain science, as well as some clinical settings.

New imaging technologies are also an active area of research in ophthalmology. Garaci et al. reported that fractional anisotropy (FA), a parameter of DTI, decreased in the early stage (MD > -6 dB) of glaucoma, while apparent diffusion coefficient (ADC) imaging was a sensitive indictor of progression in later stages of glaucoma (MD < -12 dB). [12] We have previously shown that optic nerve MRI parameters have differing correlations with the visual field, RNFL thickness, and visual acuity, suggesting that the cross-sectional area (CSA) of the optic nerve is a very promising biomarker for the objective examination of glaucoma. [13] In addition, retinotopic alteration of the visual cortex can be revealed by VBM. Differences in GMD between patients with age-related macular degeneration (AMD) and glaucoma show that in AMD, atrophy occurs in the occipital pole of the visual cortex, while in glaucoma, atrophy occurs in the medial aspect of the occipital lobe. [14] Advantageously, the brain and optic nerve are larger organs than the retina, making changes related to eye disease easier to detect. Therefore, MRI may have many potentially beneficial roles in the objective evaluation of glaucoma. However, it remains unclear whether there is a direct relationship between optic nerve degeneration and the visual cortex of the brain, which is responsible for higher-order visual function.

In this study, we utilized MRI to develop a new type of objective glaucoma examination. Our technique used recently introduced technologies, including VBM and SPM. We targeted various tissues in the visual pathway, including the optic nerve and different areas of the visual cortex (Brodmann areas [BA] 17, 18, and 19), and compared the results in normal subjects and glaucoma patients. We aimed to measure changes in the visual cortex, which is responsible for higher-order visual function, and investigate the potential of MRI as an objective technique for the examination of glaucoma.

## Material and methods

### Inclusion criteria

This prospective, cross-sectional study comprised a total of 51 eyes (20 control and 31 OAG patients) (Table 1). The inclusion criteria were: (1) a diagnosis of OAG, including primary open-angle glaucoma (POAG) or normal-tension glaucoma (NTG); (2) absence of high myopia (spherical equivalent refraction error > -8.00 diopters); and (3) a glaucomatous visual field meeting the Anderson-Patella classification. [15] The exclusion criteria were: (1) concomitant ocular disease other than OAG; (2) systemic disease affecting the visual field; (3) intraocular surgery; and (4) cataracts greater than grade 1 of the Emery-Little classification.

**Table 1:** Demographic data for this study.

	Normal (n = 20)			OAG (n = 31)			*P* value
Age	54.9	±	9.8	55.9	±	10.7	NS
Male: female	10	:	10	9	:	22	NS
Axial length (mm)	24.6	±	1.3	25.3	±	1.2	NS
IOP (mm Hg)	14.7	±	2.6	16.5	±	4.0	NS
MD (dB)	0.21	±	0.88	-8.0	±	6.82	< 0.01
cpRNFLT (μm)	111.0	±	7.5	86.1	±	11.8	< 0.01

The baseline clinical parameters recorded for each patient were age, gender and refractive error. Baseline best-corrected visual acuity was measured with a standard Japanese decimal visual acuity chart, and converted to logarithm of the minimum angle of resolution (logMAR) units for statistical analysis. IOP was measured with Goldman applanation tonometry at the time of the initial diagnosis of OAG, before the use of any medications for glaucoma.

All participants provided their written, informed consent. The study adhered to the tenets of the Declaration of Helsinki, and the protocols were approved by the Clinical Research Ethics Committee of Tohoku University Graduate School of Medicine (2015-1-560).

### Visual field analysis

Mean deviation (MD) and pattern standard deviation (PSD) were measured with the Swedish interactive threshold algorithm (SITA)-standard strategy of the 24–2 program of the Humphrey field analyzer (HFA; Carl Zeiss Meditec, Dublin, California, USA). Only MD and PSD measurements that were considered reliable (<20% fixation errors, <15% false-positive results, and <33% false-negative results) were used. The patients were divided into three groups based on the progression of their visual field defects: mild, MD > −6.0 dB; moderate, MD between −6.0 and −12.0 dB; and severe, MD < −12.0 dB.

### Regional cpRNFLT measurements and 3D OCT macular mapping

CpRNFLT was measured with 3D OCT-2000 software (version 8.00; Topcon Corporation, Tokyo, Japan). Twelve circular scans (3.46 mm in diameter) were taken, centered on the optic disc. The supplied software then determined cpRNFLT over the entire circumference of the optic disc. Images with quality less than 70 were excluded.

### Examination with MRI

#### Image acquisition

MRI examinations were performed with a 3-T scanner (Achieva Intera 3T; Philips Healthcare, Quasar Dual, the Netherlands) with an eight-element head coil. Coronal thin-sectional T2-weighted images (for calculation of optic nerve CSA) were acquired with the following imaging parameters: 5701/90 msec (repetition time/echo time), 200 × 200 field of view, and 1.2-mm section thickness with no intersection gap.

A 3-dimensional magnetization-prepared rapid acquisition gradient echo (MPRAGE) structural image was acquired using a T1-weighted magnetization sequence (repetition time [TR], 8.70 ms; echo time [TE], 3.1 ms; 8° flip angle; field of view [FOV], 256 × 256 × 180 mm; and voxel size, 0.7 × 0.7 × 0.7 mm^3^).

#### Image analysis

**Fig 1:**
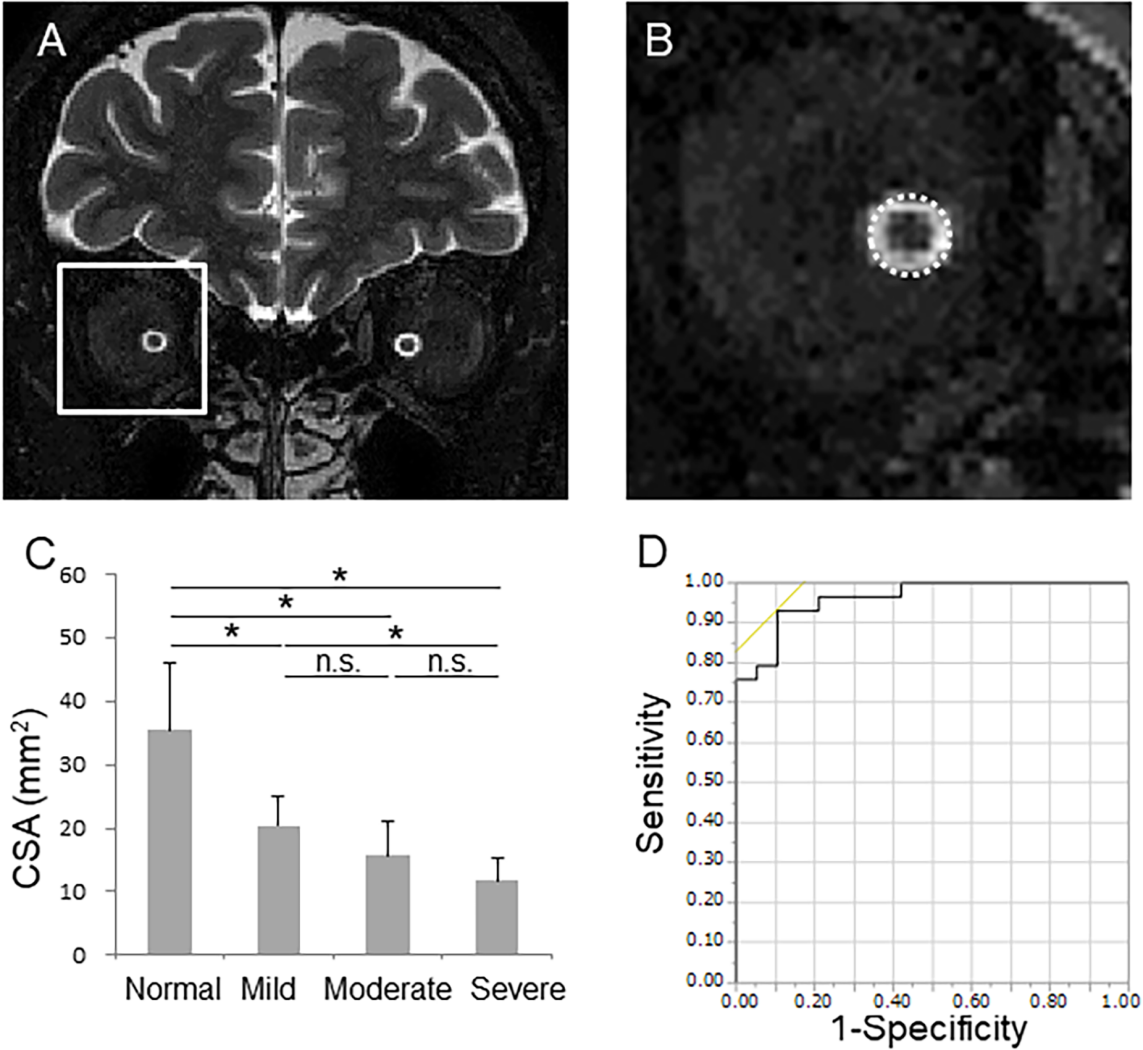
The potential of optic nerve cross-section area (CSA) in glaucoma management. (A) Coronal sections of the optic nerve region, showing the region of interest centered on the optic nerve between the optic disc and the chiasm, in a T2-weighted MRI image. (B) Higher magnification image of the white square in A. The circle indicates the optic nerve and the black circular area indicates the optic nerve. (C) Bar chart showing optic nerve CSA at different glaucoma stages. * indicates a significant difference (P<0.05). n.s.: not significant. (D) Receiver operating characteristic curve (ROC) analysis of optic nerve CSA for differentiating normal and glaucoma subjects.

Optic nerve CSA was measured in T2-weighed MRI images. Cross-sectional analysis of the optic nerve was performed using a semi-automated 3D segmentation tool (ITK-SNAP version 3.1). [16] Optic nerve CSA was determined in a region of interest (ROI) centered on the optic nerve between the optic disc and the chiasm. (Fig 1A and 1B) Normalized regional GMD was measured in the visual cortex with a volume-of-interest-based analysis.

Pre-processing of the 3D-T1 structural images was performed using Statistical Parametric Mapping software (SPM8; Wellcome Department of Cognitive Neurology, London, UK) implemented in MATLAB (Mathworks Inc., Natick, MA, USA). Using the new segmentation algorithm implemented in SPM8, the T1-weighted structural images were segmented into six types of tissue. This process used a gray matter tissue probability map (TPM), derived from maps implemented in the software, to set the signal intensity (i.e., gray matter tissue probability of the default tissue gray matter TPM + white matter tissue probability of the default TPM) of voxels <0.25 to 0. The segmentation in this study used the default parameters, with the exception of affine regularization, which used the International Consortium for Brain Mapping (ICBM) template for the brains of East Asian subjects.

We then used a diffeomorphic anatomical registration through exponentiated lie algebra (DARTEL) registration process, which is implemented in SPM8. During this process, DARTEL-imported images of the six gray matter TPMs (created using the abovementioned new segmentation process) were spatially normalized to the Montreal Neurological Institute (MNI) space to obtain images with 0.7 × 0.7 × 0.7 mm^3^ voxels. Subsequently, all images were smoothed by convolving them with an 8-mm full width at half-maximum (FWHM) isotropic Gaussian kernel, producing the final GMD maps.

#### Voxel-based morphometric analysis

Next, we performed an anatomical volume-of-interest (VOI) analysis, [17] setting the VOI at the visual cortex in BA 17, 18, and 19, as defined by the Wake Forest University Health Sciences Medical Center: Pickatlas. For both the patient and control groups, we calculated mean GMD values from identical VOIs in each subject.

### Statistical analysis

We used logistic regression analysis to measure the ability of optic nerve CSA to differentiate normal and OAG subjects. We also used the Kruskal-Wallis test and the Steel-Dwass test to compare data in the normal and OAG subjects, and to determine differences between each glaucoma stage. We performed a receiver operating characteristic curve (ROC) analysis to determine the power of CSA to discriminate between normal and OAG subjects. We compared GMD in normal and OAG subjects and investigated the relationship between CSA and GMD in BA 17, 18, and 19. Finally, we used Spearman's correlation analysis to determine the correlation between optic nerve CSA and the results of a functional examination (HFA MD and cpRNFLT), and the correlation between CSA and GMD in BA 17, 18, and 19. The significance level was set at p < 0.05. The statistical analysis was performed with JMP software (Pro version 13.1.0, SAS Institute Japan Inc., Tokyo, Japan).

## Results

Thirty-one OAG patients were divided into three groups (mild: 13, moderate: 9, severe: 9). There were no significant differences in age, axial length or IOP between these groups. The average age was 55.2 ± 11.5 years in the mild glaucoma group, 51.1 ± 9.4 in the moderate glaucoma group, and 61.6 ± 7.0 in the severe glaucoma group. Average axial length was 25.2 ± 1.3 mm in the mild glaucoma group, 25.3 ± 1.3 in the moderate glaucoma group, and 25.3 ± 1.0 in the severe glaucoma group. Baseline IOP was 15.1 ± 1.7 mmHg in the mild glaucoma group, 14.0 ± 2.3 in the moderate glaucoma group, and 15.7 ± 5.8 in the severe glaucoma group. HFA-measured MD was -2.61 ± 1.44 dB in the mild glaucoma group, -8.98 ± 1.57 in the moderate glaucoma group, and -19.2 ± 6.15 in the severe glaucoma group (p < 0.01). Average cpRNFLT was 91.4 ± 7.4 μm in the mild glaucoma group, 84.1 ± 10.3 in the moderate glaucoma group, and 76.1 ± 11.9 in the severe glaucoma group (p < 0.01).

**Fig 2:**
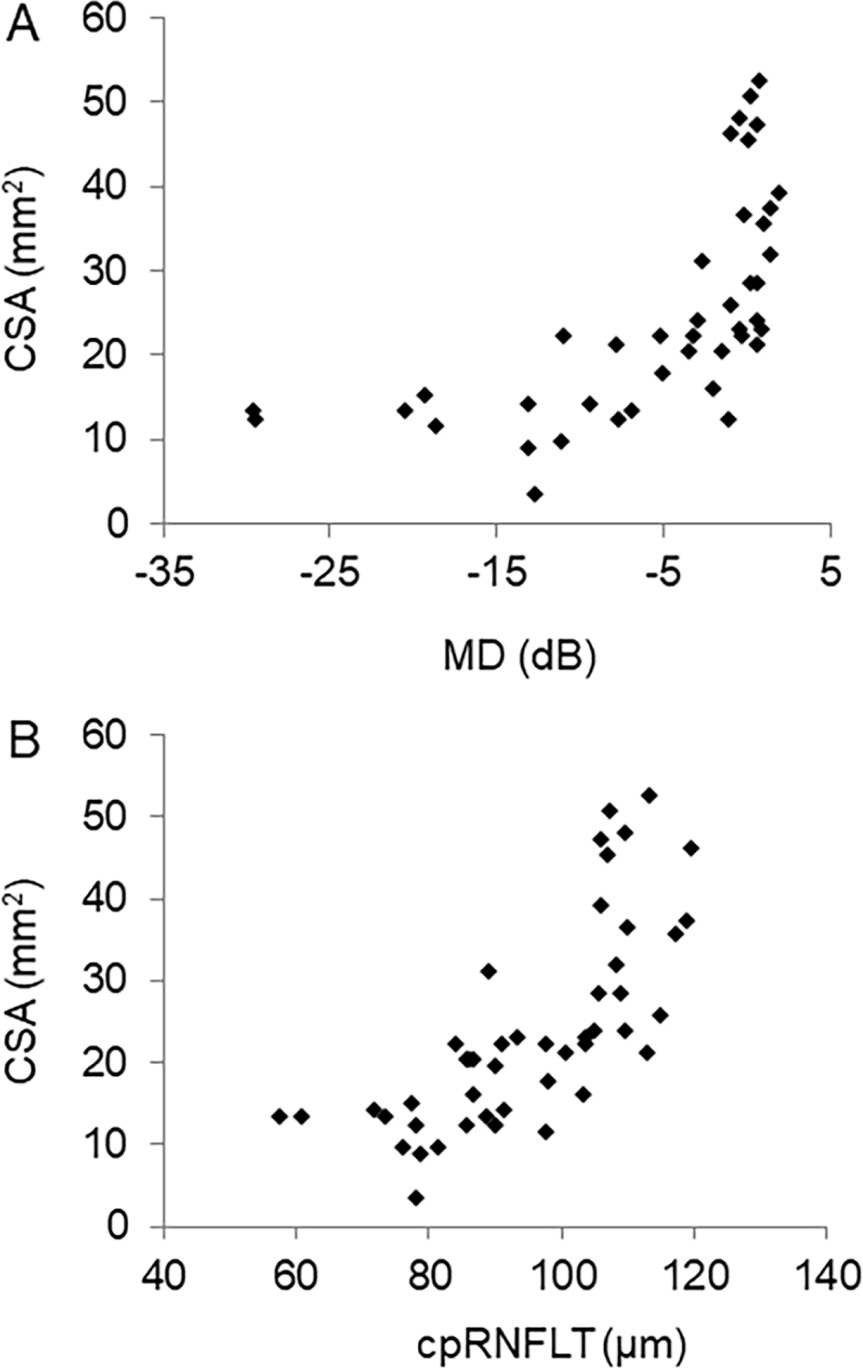
Correlation between optic nerve CSA and glaucoma severity. Scatter graph showing the relationship between CSA and HFA MD (A) and cpRNFLT (B).

Firstly, we investigated the ability of optic nerve CSA to evaluate glaucoma severity. Optic nerve CSA was significantly lower in the glaucoma patients (16.4 ± 5.8 mm^2^, p < 0.001) than the control subjects (35.5 ± 10.4 mm^2^). Optic nerve CSA at different OAG stages was as follows: 20.4 ± 4.8 mm^2^ in mild glaucoma, 15.7 ± 5.43 mm^2^ in moderate glaucoma, and 11.8 ± 3.6 mm^2^ in severe glaucoma (all p < 0.001) (Fig 1C). The ability of optic nerve CSA to distinguish normal and glaucoma subjects was excellent (AUC: 0.96, sensitivity: 93%, specificity: 90%, cut-off value: 23.2 mm^2^) (Fig 1D). There were significant correlations between CSA and cpRNFLT (r = 0.82, p < 0.001) and HFA MD (r = 0.79, p < 0.001) (Fig 2). These findings suggest that CSA is an excellent structural biomarker of glaucoma severity.

**Fig 3:**
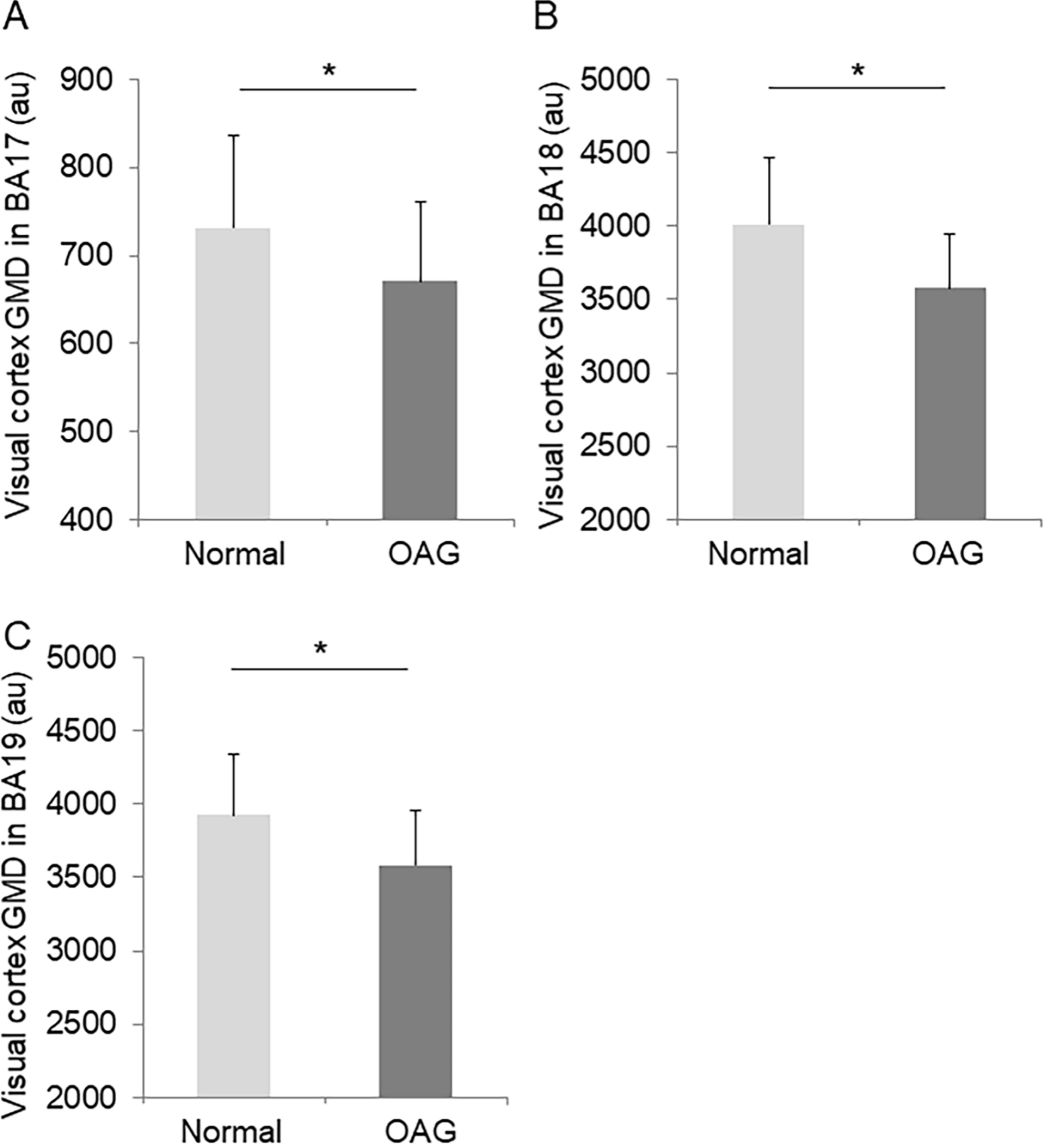
Changes in visual cortex GMD in glaucoma. Bar chart showing visual cortex GMD in normal subjects and patients with OAG in BA 17 (A), BA 18 (B), and BA 19 (C).

Next, we investigated changes in the visual cortex in BA 17, 18, and 19. We found that GMD was significantly different in the control and OAG subjects in BA 17 (controls: 731.3 ± 104.06, OAG: 670.99 ± 89.98, p = 0.030), BA 18 (control: 4011.97 ± 460.05, OAG: 3577.49 ± 363.00, p = 0.003), and BA 19 (control: 3924.13 ± 417.50, OAG: 3586.37 ± 373.76, p = 0.005) (Fig 3).

**Fig 4:**
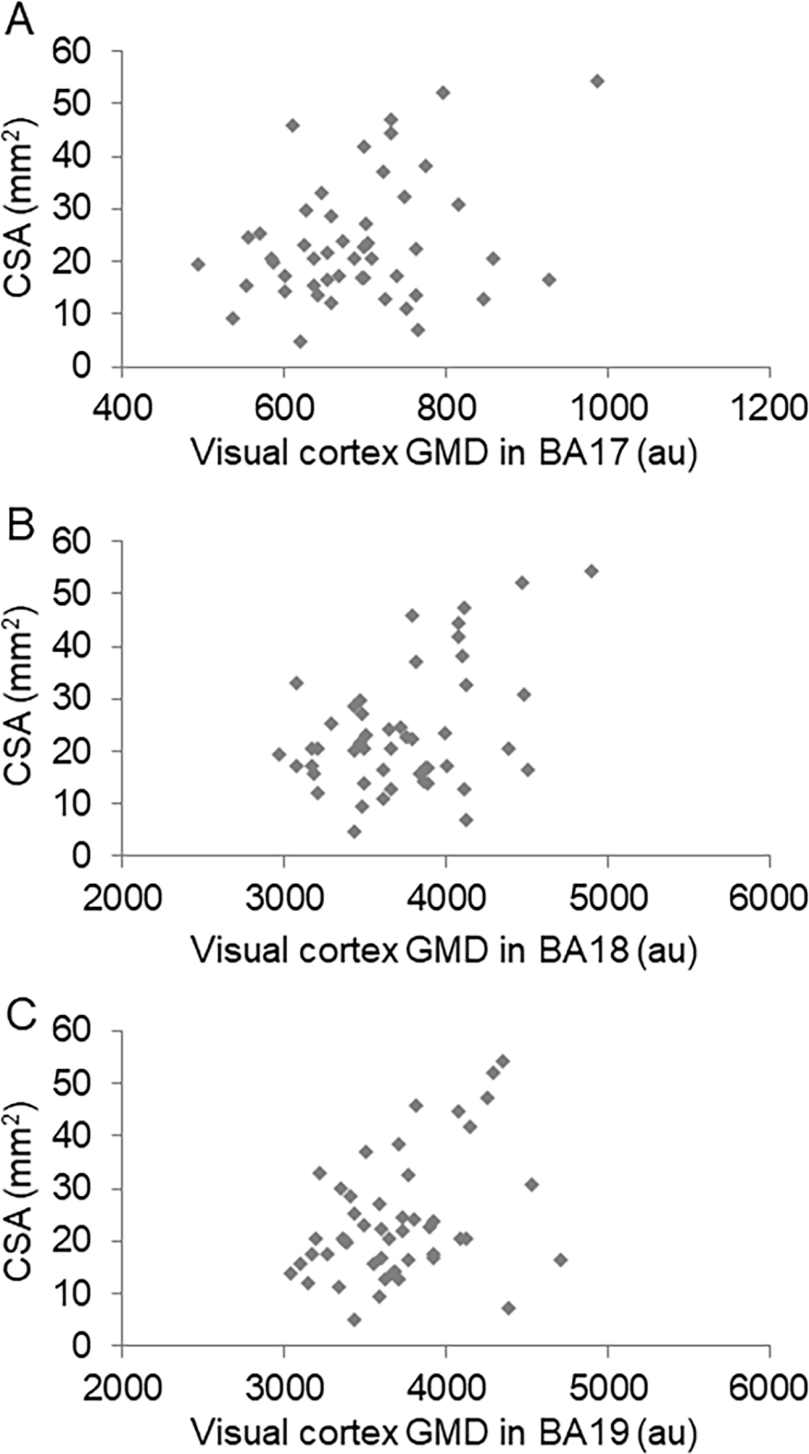
Correlation between optic nerve CSA and visual cortex GMD. Scatter graph showing the relationship between optic nerve CSA and visual cortex GMD in BA 17 (A), BA 18 (B), and BA 19 (C).

Finally, we investigated the association between visual cortex GMD and optic nerve CSA. We found a significant correlation between optic nerve CSA and visual cortex GMD in BA 19 (r = 0.33, p = 0.023), but not in BA 17 (r = 0.17, p = 0.237) or BA 18 (r = 0.24, p = 0.099) (Fig 4).

## Discussion

In this study, we found that optic nerve CSA achieved a high diagnostic efficiency for glaucoma, and that GMD in the visual cortex in BA 17, 18, and 19 was significantly decreased in patients with glaucoma. Additionally, GMD in BA 19, which plays a role in higher-order visual function, was significantly correlated to the level of optic nerve atrophy. These findings suggest that patients with OAG may also have higher-level brain dysfunction. Detailed, regional examination of the brain may therefore be useful for future glaucoma care.

Additional findings were that optic nerve CSA was significantly lower in glaucoma patients than in healthy subjects, that optic nerve CSA was significantly lower in patients with mild glaucoma than in controls, and that optic nerve CSA tended to decrease with the stage of glaucoma (Fig 1C). Furthermore, the diagnostic power of optic nerve CSA for distinguishing normal and glaucoma subjects was excellent (AUC: 0.96, sensitivity 93%, specificity 90%, cut-off values: 23.2 mm^2^) (Fig 1D). Our findings on atrophy of the optic nerve in glaucoma are consistent with previous reports. [18,19] We also found that conventional parameters of glaucoma severity, such as HFA MD (r = 0.79, p < 0.001) and cpRNFLT (r = 0.82, p < 0.001), were correlated with optic nerve CSA, with cpRNFLT having a particularly high association. Generally, thinning of the RNFLT precedes deterioration of the visual field. The high correlation we found between optic nerve CSA and cpRNFLT suggests that optic nerve CSA is a good indicator of glaucomatous structural damage, and that it is consistent with cpRNFLT. In an experimental primate model of glaucoma, early changes following the induction of ocular hypertension have shown that dephosphorylation of the neurofilaments occurs in the optic nerve, [20] and in a mouse model of ocular hypertension, the area of the optic nerve immediately decreases. [21] These findings suggest that MRI-measured optic nerve CSA could be an early structural biomarker suitable for objective glaucoma evaluation.

### Evaluation of brain visual cortex GMD in glaucoma patients

We recruited age-matched normal controls and OAG patients and compared changes to their brain in MRI scans. We found that GMD in three areas (BA 17, BA 18, and BA 19) decreased in OAG patients. The decrease in BA 17, which contains the primary visual cortex (V1) has already been reported by other groups. [22,23] Furthermore, functional MRI investigation of cortical activity in the human V1 has shown that it is significantly altered in POAG subjects, in a manner consistent with damage to the optic disc. [24,25] Thus, in order to discuss brain damage in glaucoma, we need to pay attention to two basic functional connections. First, retinotopy; the connection between the retina and visual cortex is tightly maintained in the V1, but not beyond the V2. [26,27] Retinal damage shows strong corresponding changes in the visual cortex. [14] Second, after being projected to the V1, visual information is projected to two major pathways. Leading to the upper central nerve, the ventral pathway connects to the inferotemporal cortex, which processes color and shape information, and the dorsal pathway connects to the parietal lobe, which processes spatial and motion information. Furthermore, the occipital lobe, cerebellum, and hippocampus have also been demonstrated to be damaged in glaucoma. [11] Thus, glaucoma can be considered a widespread neurodegenerative disease.

### Our choice of optic nerve CSA as a comparative parameter for GMD

In this study, we found that the strength of the correlation between optic nerve CSA and visual cortex GMD differed in different regions. Previous reports have used cpRNFLT (measured with OCT) or MD (measured with HFA) as parameters of the severity of glaucoma, but we chose to use optic nerve CSA because it showed good correlations with other parameters. In fact, parameters such as cpRNFLT and MD each have their own disadvantages. MD is useful in the severe stages of glaucoma, but less so in the earlier stages, because of its poor sensitivity and the fact that it does not reflect the entire visual field (for example, the HFA 24–2 test measures 24 degrees temporally and 30 degrees nasally, and tests 54 points.) On the other hand, cpRNFLT is not a sensitive indicator in severe glaucoma, due to the floor effect. Additionally, elongated axial length, such as in myopia, can bias cpRNFLT results. By contrast, the optic nerve is closer to the visual cortex than these intraocular parameters. Thus, we chose to compare optic nerve CSA and visual cortex GMD in this study because of these characteristics.

### Correlation between CSA and GMD in BA19

We detected a significant correlation between CSA and GMD in BA 19, but not in BA 17. Furthermore, the relationship between CSA and GMD in BA 18 was marginal. Details on the function of BA 19, which contains the third visual cortex (V3), remain unclear, but BA 19 is thought to play a role in the processing of global motion and sensitivity to orientation, depth, movement, stereoacuity, and color. Coherent motion has been shown to induce the greater activation of V3, but not V1. [28] In glaucoma, decreased function of cortically processed global motion, global form [29] and stereoacuity [30] have been reported. These higher-order visual dysfunctions are consistent with corresponding decreases in GMD in BA 19, as we observed here in glaucoma patients.

In contrast with BA 19, all visual information enters the V1 (i.e., BA 17). Retinotopy (the mapping of visual input from the retina to neurons) is strongly maintained in V1. The mapping becomes more complicated as other signals join beyond the V2 (i.e., in BA 18). [27] The central 10 degrees of the visual field are represented by 50% of the posterior striate cortex (in V1), and the central 30 degrees are represented by 80% of this cortex, which suggests that the greater part of V1 is related to the central visual field. [31] Generally, the Bjerrum area is susceptible to glaucomatous damage, and the central visual field tends to be preserved until the severe stages of glaucoma. Tight retinotopy mapping may explain why GMD in BA 17 was not significantly associated with CSA in this study. Moreover, it may explain the marginal relationship we found between CSA and GMD in BA 18 (i.e., V2). Therefore, in glaucoma patients, higher-order visual dysfunction in BA 18 and 19 may be associated with the severity of glaucoma, suggesting that a detailed regional assessment of the visual cortex with MRI would help improve the objective evaluation of glaucoma severity.

### Additional advantages of MRI in glaucoma

Our findings that optic nerve CSA and visual cortex GMD were related to glaucoma severity suggests an additional advantage in certain conditions. These new MRI parameters are particularly valuable in conditions that cause conventional tests, such as visual field tests and OCT, to be difficult to carry out or not reliable. These conditions include (1) opacity of the intermediate optic media, such as corneal opacity, uveitis, or often cataract, (2) difficult patients (e.g., children, or patients with dementia or mental retardation), or (3) abnormal appearance of the optic nerve head (such as in high myopia or congenital optic nerve anomalies). In Asia, myopia has a high prevalence and is a risk factor for glaucoma. [32] Myopic deformity of the optic nerve head often makes it difficult to diagnose glaucoma. Thus, the high discriminative power of MRI-measured optic nerve CSA may be beneficial in the clinic. Another benefit of this MRI-based method is that it should enable us, in the near future, to perform glaucoma screening in patients who are already undergoing MRI as part of standard health checks. While MRI also has disadvantages, such as being expensive and time-consuming, its advantages are enormous, at least in specific situations. In summary, we consider that MRI analysis of the optic nerve may have value as an objective method to assess glaucomatous degeneration in clinical practice.

### Limitations

We would like to describe the limitations of this study. First, we included only adult patients of a single ethnicity (Japanese). We also included only a small number of participants (51), which may have affected the power of the significance and subclass analyses. Moreover, the association between optic nerve CSA and GMD in BA 18 was marginal (p = 0.099). Furthermore, POAG may have different types of pathogenesis at different stages. [33] Dissimilar changes in the right and left hemispheres, as well as in different cortical areas, have been reported. [31] Thus, additional investigation of these factors is needed, with multiple regression analysis and a larger number of subjects. A second limitation was our reliance on two well-trained radiologists to interpret the MR images and carefully exclude patients, with consideration of their history, who had obvious brain disease or other major diagnostic systemic diseases that lead to brain atrophy. However, patients with subclinical disease were not excluded from this study. It has been reported that low blood pressure, diabetes, sleep apnea syndrome, migraine, depression and aging [34,35] can influence brain volume, and other factors, such as oxidative stress, medicine use, and alcohol use may influence the structure of the brain. Furthermore, these risk factors are also related to the presence of OAG. [36] Thus, we cannot exclude the possibility that some risk factors simultaneously induced both brain atrophy and glaucoma, which could have affected our results. More detailed regional analysis is therefore needed.

## Conclusion

In conclusion, we found that optic nerve CSA, measured by MRI, showed good diagnostic potential, at the same level as conventional parameters, for evaluating glaucoma. Furthermore, we are the first to find that visual cortex GMD in BA 17, BA 18, and BA 19 significantly decreases in patients with OAG, and that among these areas of the brain, optic nerve CSA had the strongest correlation with BA 19. These new, emerging techniques based on MRI are non-invasive and objective, and may therefore have clinical value equal to conventional examinations, such as visual field testing and OCT, for assessing glaucomatous degeneration. Furthermore, MRI testing has advantages in cases in which subjective tests for glaucoma are difficult, and should allow glaucoma screening in patients undergoing general health checks. MRI analysis of the optic nerve and visual cortex, which governs higher-order visual functions, is potentially very promising for the objective examination of patients with glaucoma. MRI analysis may also open new avenues of research into the relationship between structure and function in the brain, and enable the development of new functional tests for glaucoma in the near future.

## Supporting information

S1 File Supplementary data of this study. (XLSX)

## References

[1] Weinreb RN, Khaw PT. Primary open-angle glaucoma. Lancet. 2004;363: 1711–1720. doi: 10.1016/S0140-6736(04)16257-0 15158634 10.1016/S0140-6736(04)16257-0

[2] Leske MC, Heijl A, Hyman L, Bengtsson B, Dong L, Yang Z, et al Predictors of long-term progression in the early manifest glaucoma trial. Ophthalmology. 2007;114: 1965–1972. doi: 10.1016/j.ophtha.2007.03.016 17628686 10.1016/j.ophtha.2007.03.016

[3] Yokoyama Y, Maruyama K, Konno H, Hashimoto S, Takahashi M, Kayaba H, et al Characteristics of patients with primary open angle glaucoma and normal tension glaucoma at a university hospital: a cross-sectional retrospective study. BMC Res Notes. 2015;8: 360 doi: 10.1186/s13104-015-1339-x 26286038 10.1186/s13104-015-1339-x PMC4541728

[4] Iwase A, Suzuki Y, Araie M, Yamamoto T, Abe H, Shirato S, et al The prevalence of primary open-angle glaucoma in Japanese: the Tajimi Study. Ophthalmology. 2004;111: 1641–1648. doi: 10.1016/j.ophtha.2004.03.029 15350316 10.1016/j.ophtha.2004.03.029

[5] Ernest PJ, Schouten JS, Beckers HJ, Hendrikse F, Prins MH, Webers CA. An evidence-based review of prognostic factors for glaucomatous visual field progression. Ophthalmology. 2013;120: 512–519. doi: 10.1016/j.ophtha.2012.09.005 23211636 10.1016/j.ophtha.2012.09.005

[6] Gupta N, Ang LC, Noel de Tilly L, Bidaisee L, Yucel YH. Human glaucoma and neural degeneration in intracranial optic nerve, lateral geniculate nucleus, and visual cortex. Br J Ophthalmol. 2006;90: 674–678. doi: 10.1136/bjo.2005.086769 16464969 10.1136/bjo.2005.086769 PMC1860237

[7] Gupta N, Greenberg G, de Tilly LN, Gray B, Polemidiotis M, Yucel YH. Atrophy of the lateral geniculate nucleus in human glaucoma detected by magnetic resonance imaging. Br J Ophthalmol. 2009;93: 56–60. doi: 10.1136/bjo.2008.138172 18697810 10.1136/bjo.2008.138172 PMC2605243

[8] Yucel YH, Gupta N. Paying attention to the cerebrovascular system in glaucoma. Can J Ophthalmol. 2008;43: 342–346. doi: 10.3129/i08-059 18493275 10.3129/i08-059

[9] Gupta N, Yucel YH. Glaucoma as a neurodegenerative disease. Curr Opin Ophthalmol. 2007;18: 110–114. doi: 10.1097/ICU.0b013e3280895aea 17301611 10.1097/ICU.0b013e3280895aea

[10] Nucci C, Martucci A, Cesareo M, Mancino R, Russo R, Bagetta G, et al Brain involvement in glaucoma: advanced neuroimaging for understanding and monitoring a new target for therapy. Curr Opin Pharmacol. 2013;13: 128–133. doi: 10.1016/j.coph.2012.08.004 22981808 10.1016/j.coph.2012.08.004

[11] Frezzotti P, Giorgio A, Motolese I, De Leucio A, Iester M, Motolese E, et al Structural and functional brain changes beyond visual system in patients with advanced glaucoma. PLoS One. 2014;9: e105931 doi: 10.1371/journal.pone.0105931 25162716 10.1371/journal.pone.0105931 PMC4146554

[12] Garaci FG, Bolacchi F, Cerulli A, Melis M, Spano A, Cedrone C, et al Optic nerve and optic radiation neurodegeneration in patients with glaucoma: in vivo analysis with 3-T diffusion-tensor MR imaging. Radiology. 2009;252: 496–501. doi: 10.1148/radiol.2522081240 19435941 10.1148/radiol.2522081240

[13] Omodaka K, Murata T, Sato S, Takahashi M, Tatewaki Y, Nagasaka T, et al Correlation of magnetic resonance imaging optic nerve parameters to optical coherence tomography and the visual field in glaucoma. Clin Exp Ophthalmol. 2014;42: 360–368. doi: 10.1111/ceo.12237 24119065 10.1111/ceo.12237

[14] Boucard CC, Hernowo AT, Maguire RP, Jansonius NM, Roerdink JB, Hooymans JM, et al Changes in cortical grey matter density associated with long-standing retinal visual field defects. Brain. 2009;132: 1898–1906. doi: 10.1093/brain/awp119 19467992 10.1093/brain/awp119 PMC2702836

[15] Anderson DR, Patella VM (1999) Automated Static Perimetry: Mosby.

[16] Yushkevich PA. User-guided 3D active contour segmentation of anatomical structures: significantly improved efficiency and reliability. NeuroImage. 2006;31: 1116–1128. doi: 10.1016/j.neuroimage.2006.01.015 16545965 10.1016/j.neuroimage.2006.01.015

[17] Ashburner J, Friston KJ. Voxel-based morphometry−the methods. Neuroimage. 2000;11: 805–821. doi: 10.1006/nimg.2000.0582 10860804 10.1006/nimg.2000.0582

[18] Kashiwagi K, Okubo T, Tsukahara S. Association of magnetic resonance imaging of anterior optic pathway with glaucomatous visual field damage and optic disc cupping. J Glaucoma. 2004;13: 189–195. 15118461 10.1097/00061198-200406000-00003

[19] Ramli NM, Sidek S, Rahman FA, Peyman M, Zahari M, Rahmat K, et al Novel use of 3T MRI in assessment of optic nerve volume in glaucoma. Graefes Arch Clin Exp Ophthalmol. 2014;252: 995–1000. doi: 10.1007/s00417-014-2622-6 24770532 10.1007/s00417-014-2622-6

[20] Kashiwagi K, Ou B, Nakamura S, Tanaka Y, Suzuki M, Tsukahara S. Increase in dephosphorylation of the heavy neurofilament subunit in the monkey chronic glaucoma model. Invest Ophthalmol Vis Sci. 2003;44: 154–159. 12506068 10.1167/iovs.02-0398

[21] Nakazawa T, Nakazawa C, Matsubara A, Noda K, Hisatomi T, She H, et al Tumor necrosis factor-alpha mediates oligodendrocyte death and delayed retinal ganglion cell loss in a mouse model of glaucoma. J Neurosci. 2006;26: 12633–12641. doi: 10.1523/JNEUROSCI.2801-06.2006 17151265 10.1523/JNEUROSCI.2801-06.2006 PMC6674838

[22] Chen WW, Wang N, Cai S, Fang Z, Yu M, Wu Q, et al Structural brain abnormalities in patients with primary open-angle glaucoma: a study with 3T MR imaging. Invest Ophthalmol Vis Sci. 2013;54: 545–554. doi: 10.1167/iovs.12-9893 23258150 10.1167/iovs.12-9893

[23] Zhou W, Muir ER, Chalfin S, Nagi KS, Duong TQ. MRI Study of the Posterior Visual Pathways in Primary Open Angle Glaucoma. J Glaucoma. 2017;26: 173–181. doi: 10.1097/IJG.0000000000000558 27661989 10.1097/IJG.0000000000000558 PMC5288289

[24] Duncan RO, Sample PA, Weinreb RN, Bowd C, Zangwill LM. Retinotopic organization of primary visual cortex in glaucoma: a method for comparing cortical function with damage to the optic disk. Invest Ophthalmol Vis Sci. 2007;48: 733–744. doi: 10.1167/iovs.06-0773 17251472 10.1167/iovs.06-0773

[25] Duncan RO, Sample PA, Weinreb RN, Bowd C, Zangwill LM. Retinotopic organization of primary visual cortex in glaucoma: Comparing fMRI measurements of cortical function with visual field loss. Prog Retin Eye Res. 2007;26: 38–56. doi: 10.1016/j.preteyeres.2006.10.001 17126063 10.1016/j.preteyeres.2006.10.001 PMC1940234

[26] Wandell BA, Brewer AA, Dougherty RF. Visual field map clusters in human cortex. Philos Trans R Soc Lond B Biol Sci. 2005;360: 693–707. doi: 10.1098/rstb.2005.1628 15937008 10.1098/rstb.2005.1628 PMC1569486

[27] Wandell BA, Dumoulin SO, Brewer AA. Visual field maps in human cortex. Neuron. 2007;56: 366–383. doi: 10.1016/j.neuron.2007.10.012 17964252 10.1016/j.neuron.2007.10.012

[28] Braddick OJ, O'Brien JM, Wattam-Bell J, Atkinson J, Hartley T, Turner R. Brain areas sensitive to coherent visual motion. Perception. 2001;30: 61–72. doi: 10.1068/p3048 11257978 10.1068/p3048

[29] McKendrick AM, Badcock DR, Morgan WH. The detection of both global motion and global form is disrupted in glaucoma. Invest Ophthalmol Vis Sci. 2005;46: 3693–3701. doi: 10.1167/iovs.04-1406 16186351 10.1167/iovs.04-1406

[30] Lakshmanan Y, George RJ. Stereoacuity in mild, moderate and severe glaucoma. Ophthalmic Physiol Opt. 2013;33: 172–178. doi: 10.1111/opo.12021 23297812 10.1111/opo.12021

[31] Horton JC, Hoyt WF. The representation of the visual field in human striate cortex. A revision of the classic Holmes map. Arch Ophthalmol. 1991;109: 816–824. 2043069 10.1001/archopht.1991.01080060080030

[32] Suzuki Y, Iwase A, Araie M, Yamamoto T, Abe H, Shirato S, et al Risk factors for open-angle glaucoma in a Japanese population: the Tajimi Study. Ophthalmology. 2006;113: 1613–1617. doi: 10.1016/j.ophtha.2006.03.059 16828504 10.1016/j.ophtha.2006.03.059

[33] Li C, Cai P, Shi L, Lin Y, Zhang J, Liu S, et al Voxel-based morphometry of the visual-related cortex in primary open angle glaucoma. Curr Eye Res. 2012;37: 794–802. doi: 10.3109/02713683.2012.683506 22631870 10.3109/02713683.2012.683506

[34] Dieleman N, Koek HL, Hendrikse J. Short-term mechanisms influencing volumetric brain dynamics. Neuroimage Clin. 2017;16: 507–513. doi: 10.1016/j.nicl.2017.09.002 28971004 10.1016/j.nicl.2017.09.002 PMC5609861

[35] Gudmundsson LS, Scher AI, Sigurdsson S, Geerlings MI, Vidal JS, Eiriksdottir G, et al Migraine, depression, and brain volume: the AGES-Reykjavik Study. Neurology. 2013;80: 2138–2144. doi: 10.1212/WNL.0b013e318295d69e 23700334 10.1212/WNL.0b013e318295d69e PMC3716352

[36] Nakazawa T. Ocular Blood Flow and Influencing Factors for Glaucoma. Asia Pac J Ophthalmol (Phila). 2016;5: 38–44. 26886118 10.1097/APO.0000000000000183

